# Early outcomes of contaminated midline incisional hernia repair with mesh suture

**DOI:** 10.1007/s10029-025-03422-8

**Published:** 2025-08-04

**Authors:** Megan M. Perez, Taaha Hassan, Mehul Mittal, May Li, Kazimir Bagdady, Paige N. Hackenberger, Gregory A. Dumanian, Michael Shapiro

**Affiliations:** Department of Surgery, Northwestern Feinberg School of Medicine, 676 N. St. Clair Street, Suite 2320, Chicago, IL 60611 USA

**Keywords:** Ventral hernia, Incisional hernia, Contaminated hernia repair, Suture repair, Mesh repair, Recurrence

## Abstract

**Purpose:**

Mesh suture is a novel reinforcement construct designed to provide enhanced mechanical support during midline fascial closure in comparison to conventional sutures while minimizing tissue dissection and foreign body burden in comparison to use of a planar mesh. Its use in contaminated fields remains understudied. This study evaluates the early clinical outcomes following mesh suture closure in clean-contaminated and contaminated incisional hernia repairs.

**Methods:**

A retrospective review was conducted of patients undergoing incisional hernia repair with mesh suture closure between January 2023 and July 2024 across an academic health system. Eligible patients had clean-contaminated or contaminated wounds and underwent mesh suture implantation without planar mesh. Data included demographics, operative details, surgical site infections (SSI), surgical site occurrences (SSO), reoperations, readmissions, and hernia recurrence. Hernia recurrence-free survival was estimated using Kaplan-Meier analysis. Major complications were defined as surgical complication or reoperation within 90 days.

**Results:**

Fifty-one patients were included, with repairs performed by 22 surgeons. Most patients (62.7%) had clean-contaminated wounds. Anterior component separation was performed in 25.5% of cases. The 90-day SSI rate was 15.7%, with five patients requiring procedural intervention. The 90-day SSO rate was 23.5% and included one enterocutaneous fistula that resolved without surgical intervention. Readmission occurred in 27.4% of patients, and 9.8% underwent reoperation. Four hernia recurrences were observed (8.2%), with a 12-month recurrence-free survival of 91% and a mean recurrence-free survival of 17.3 months (95% CI: 16.5–18.1). Presence of a stoma was significantly associated with major complications (*p* = 0.041).

**Conclusion:**

Mesh suture closure was technically feasible across diverse surgical settings and demonstrated encouraging SSI and SSO rates as compared to conventional suture only and planar mesh-based repairs as reported in the literature. These findings support further investigation in prospective, comparative studies to assess long-term durability and comparative effectiveness.

**Supplementary Information:**

The online version contains supplementary material available at 10.1007/s10029-025-03422-8.

## Introduction

Optimal closure of clean-contaminated and contaminated incisional hernias remains a clinical challenge across surgical disciplines. In these settings, simple midline closure with suture may be favored due to its minimal invasiveness; however, suture-only repairs are associated with high hernia recurrence rates, reported in some series to exceed 50% [1–3]. This is partly due to focal tension at the suture–tissue interface (STI) that can lead to suture pull-through or “cheese-wiring” of the fascia. To improve durability, reinforcement with planar mesh has become the standard in clean hernia repairs and is increasingly considered in contaminated settings. However, no single approach has demonstrated consistent superiority, and the optimal reinforcement strategy in this context remains debated. Various mesh types, including permanent synthetic, absorbable synthetic, and biologic materials offer structural support and have been shown to reduce recurrence significantly as compared to suture only repairs [3–7]. However, each material has its own drawbacks.

Biologic mesh, once favored in contaminated fields due to presumed infection resistance, has shown limited long-term durability with recurrence rates exceeding 30% [3, 5, 8]. Inconsistent outcomes and high costs has driven interest towards biosynthetic and synthetic alternatives [5, 8]. Biosynthetic absorbable meshes may offer favorable short-term complications profiles compared to biologics and without the feared consequences of a permanent prosthetic mesh infection, though long-term data remains limited [7]. Permanent synthetic meshes, particularly when placed in a retrorectus position, have demonstrated promising results in select contaminated cases with SSI rate of 21%, mesh explantation rate of 4% and hernia recurrence rate of 15% [7]. While a growing body of evidence is in support of the selective use of permanent synthetic mesh, these repairs require the extensive dissection of tissue planes and may be best suited to an experienced abdominal wall surgeon, thereby limiting generalizability to a wide patient population.

**Fig. 1:**
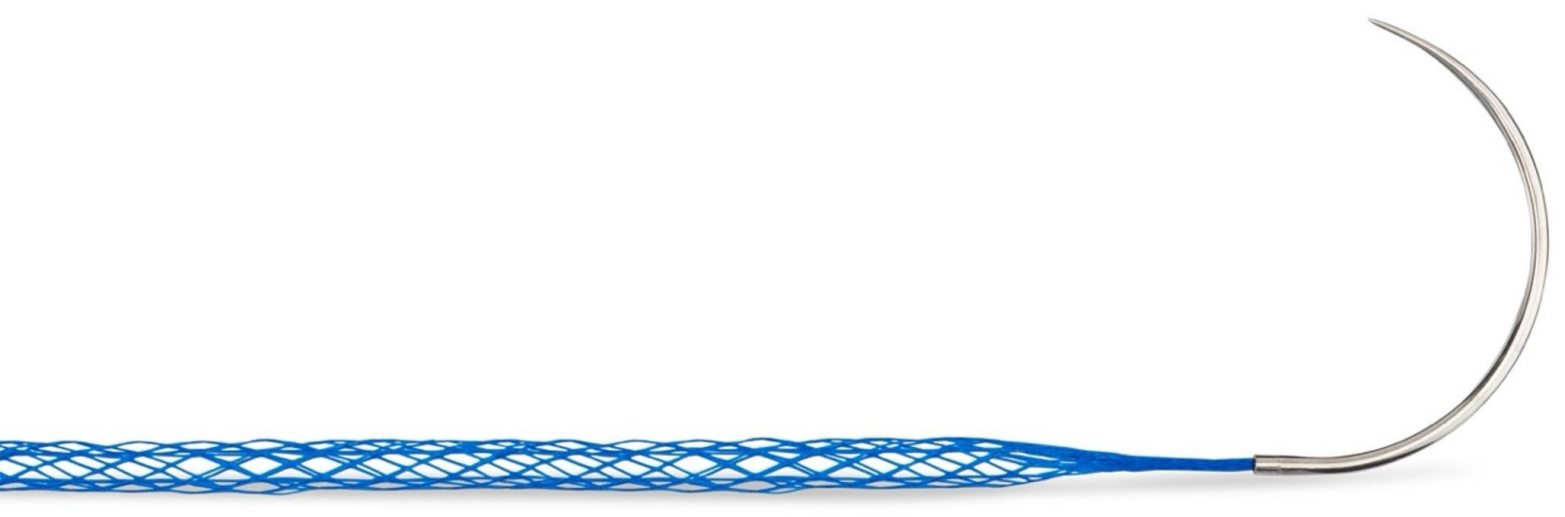
Mesh suture is composed of 18 strands of fine polypropylene filaments that are braided and bonded into a linear large-pore construct, device MSI-301 pictured

To address these limitations, mesh suture (Duramesh, Mesh Suture Inc., Chicago, IL) was developed as a potential alternative that bridges the gap between traditional suture closure and planar mesh reinforcement. Mesh suture is composed of 18 strands of fine polypropylene filaments that are braided and bonded into a large pore, linear construct, that flattens under tension, increasing contact surface area to diffuse focal tension at the STI (Fig. 1). This design may reduce local tissue trauma and the risk of suture pull-through [9]. Pre-clinical studies have shown that mesh suture resists pull-through better than standard suture in a bovine model and undergoes rapid fibrovascular incorporation, potentially contributing to early strength and infection resistance [10–12]. As such, mesh suture may represent a viable, tissue-sparing alternative to achieve a lasting abdominal closure in settings where planar mesh may not be preferred by the surgeon due to specific patient factors or clinical scenarios. 

This retrospective feasibility study evaluated the early clinical performance of mesh suture closure in clean contaminated and contaminated midline incisional hernia repair. It was hypothesized that mesh suture could provide improved early mechanical support as compared to traditional suture only repairsz, and operative advantages and complication rates as compared to planar mesh-based repairs, as reported in the literature. Short-term outcomes assessed included SSI, surgical site occurrences (SSO), reoperation, readmission, and hernia recurrence rates, as an initial evaluation of the feasibility of mesh suture in this context. Given the modest sample size and limited follow up, this study is not intended to establish comparative safety or long-term durability, but rather to provide foundational data to inform future prospective or comparative trials.

## Materials and methods

### Data collection

After Institutional Review Board (IRB) approval, a retrospective cohort study was conducted on patients who underwent mesh suture closure for contaminated midline incisional hernia repair between January 23, 2023, and July 24, 2024. Procedures were performed by multiple surgeons across a single integrated health system comprising one urban academic hospital and several affiliated community-based hospitals. Eligible patients underwent closure with mesh suture for a midline incisional hernia in the setting of clean contaminated (Centers for Disease Control [CDC] Class II) or contaminated (CDC Class III) wounds. Patients were excluded if they underwent isolated parastomal hernia repair, non-midline hernia repair, hernia repair involving planar mesh, or other types of ventral (non-incisional) hernias.

Mesh suture was used at the discretion of the operating surgeon as part of routine clinical practice. Patients did not provide additional informed consent for mesh suture use, and no surgeon received incentives or external training related to the device. Instructions for use were available, but no formal education sessions were provided. Wound bed preparation, antibiotic administration, and strategies to reduce bacterial bioburden were determined by individual surgeon preference. Data collected included patient demographics, comorbidities, surgical history, hernia characteristics, and intraoperative findings. Preoperative hernia status was determined based on documentation from physical examination, abdominal CT imaging, or operative reports. Active smoking was defined as smoking within four weeks of surgery. Contamination sources were reviewed and recorded for each case.

Follow-up was assessed through clinical documentation and imaging. Data collection was truncated at either the date of mesh suture removal or the study end date. The primary outcome was 90-day SSI rate as defined by Majumder et al. [13]. Secondary outcomes included SSOs defined according the Ventral Hernia Working Group criteria including SSI, seroma, hematoma, fascial dehiscence, non-healing wound/soft tissue breakdown, chronic mesh infections, cellulitis, suture granuloma, and enterocutaneous fistula [14]. Other outcomes included readmissions, reoperations, and hernia recurrence. Hernia recurrence was defined as documentation of a recurrent defect based on physical exam or cross-sectional imaging. Reoperations were defined as any unplanned surgical procedure at the site of mesh suture implantation. Major complications were defined as complication or reoperation occurring within 90 days.

### Operative technique

**Fig. 2:**
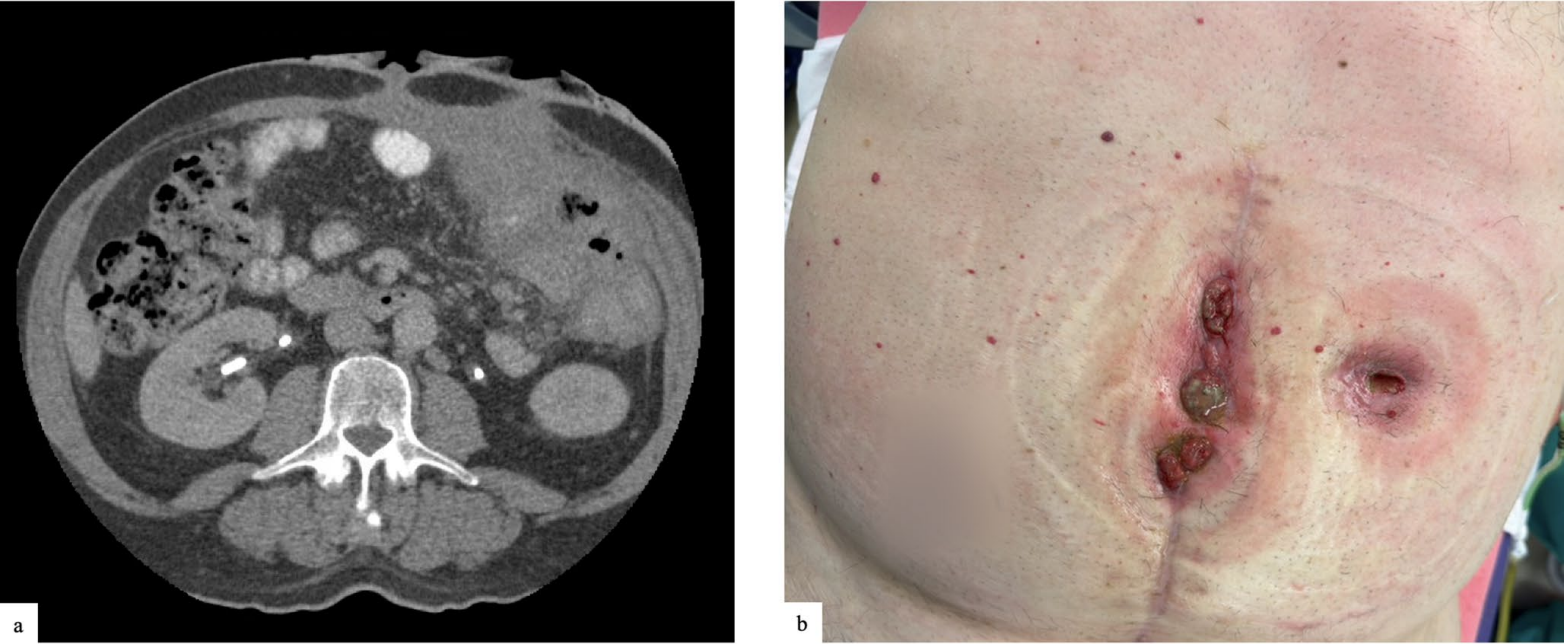
shows a 60-year-old man with a history of Crohn’s disease and multiple enterocutaneous fistulae presenting for fistula takedown, incisional hernia repair and ostomy reversal, (**a**) shows the preoperative axial CT scan with evidence of multiple fistula and significant hernia (**b**) preoperative clinical photographs of wounds

**Fig. 3:**
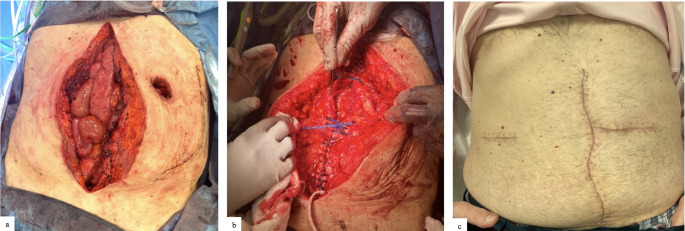
shows intraoperative mesh suture repair of patient in Fig. 2, (**a**) defect after completion of ostomy and fistula takedown and hernia sac excision, fascial edges were circumferentially cleared to healthy tissue to optimize approximation. Given significant transverse width of defect, a bilateral anterior components release was performed through a counter incision on the right and through the old ostomy site on the left, (**b**) mesh suture closure of defect in a continuous running fashion, using full-thickness, 1 cm through-and-through bites of the anterior abdominal wall with 8 mm travel between bites. The ostomy abdominal wall defect was closed vertically with mesh suture. Ostomy site skin was sharply debrided and then closed transversely to meet the midline at a “T,” (**c**) immediately postoperative, the incision on the right was used for the right anterior components separation, the ostomy site on the left was used for the left components separation and was then closed transversely

Although mesh suture closure was performed by multiple surgeons, a broadly consistent technique was utilized across cases. After complete adhesiolysis and adequate exposure, the fascial edges were circumferentially cleared to healthy tissue to optimize approximation. Mesh suture was typically placed in a continuous running fashion, using full-thickness, through-and-through bites of the anterior abdominal wall to achieve secure re-approximation of the midline. In general, surgeons took 1 cm bites of tissue after debridement to healthy wound edges and 8 mm travel between bites. The closure technique was conceptually inspired by the small-bites fascial closure principles established in the STITCH trial that emphasized the distribution of suture tension across multiple filaments spanning the fascial closure. However, due to the larger physical profile of mesh suture compared to monofilament suture, slightly larger bites as described above were employed and is based on pre-clinical porcine studies [11, 15]. Figures 2 and 3 show a clinical example of the technique most used. This technique allowed for uniform tension distribution and midline reinforcement. In cases where primary fascial closure was not feasible due to excessive tension, anterior component separation was performed using a perforator-sparing technique as detailed by Ko et al. [16]. In brief, 6–8 cm transverse counter-incisions were placed over the semilunar lines to allow for direct external oblique release without requiring development of wide skin flaps, minimizing the disruption of vascular supply to the soft tissues and skin (Fig. 3).

### Data analysis

Data analysis was performed using IBM SPSS Statistics 29. T test and chi-square were used when appropriate. Recurrence-free survival was estimated using Kaplan-Meier analysis, with time to event defined as the duration in months from the index surgery to either hernia recurrence or last follow-up. To address heavy censoring and sparse data beyond 18 months, the survival function was truncated at 18 months to reflect the period of consistent follow-up across the cohort. Patients who experienced recurrence after 18 months were censored in this analysis, and all time-to-event data were capped at 18 months for consistency. A value of *p* < 0.05 was considered statistically significant.

## Results

### Patient demographics

A total of 51 patients met inclusion criteria, with repairs performed by 22 surgeons from seven surgical subspecialties (Table 1). Most procedures (90.2%) were completed at an academic medical center. The average patient age was 62.4 (± 14.2) years, and 41.2% were male. Wound contamination was categorized as clean contaminated in 32 patients (62.7%) and contaminated in 19 patients (37.3%). Patients had undergone an average of 2.2 (± 1.6) prior abdominal surgeries, and 37.3% had at least one prior hernia repair. The most common sources of contamination were bowel resection (43.1%) and lysis of adhesions or bowel manipulation (11.7%). The remaining demographics are detailed in Table 1.

**Table 1:** Patient demographics, comorbidities, source of contamination and preoperative hernia characteristics

	*N* = 51 (%)
Age (mean, SD)	62.4 ± 14.2
Male Sex	21 (41.2)
BMI (mean, SD)	28.8 ± 7.5
Current smoker	5 (9.8)
Former smoker	26 (51.0)
Cancer	15 (29.4)
COPD	10 (19.6)
HTN	40 (78.4)
DM	12 (23.5)
ASA classification	
II	4 (7.8)
III	44 (86.3)
IV	3 (5.9)
CDC wound classification	
Clean contaminated	32 (62.7)
Contaminated	19 (37.3)
Surgeon specialty	
Colorectal	20 (39.2)
General surgery	8 (15.7)
Trauma	6 (11.8)
Urology	5 (9.8)
Gynecology	5 (9.8)
Plastic surgery	4 (7.8)
Transplant	3 (5.9)
Contamination	
Bowel resection	22 (43.1)
Ostomy reversal	3 (5.9)
Gynecologic procedure	4 (7.8)
Urologic procedure	4 (7.8)
Creation of stoma/revision	4 (7.8)
Enterotomy	1 (2.0)
Cholecystectomy	2 (3.9)
Fistula	5 (9.8)
Lysis of adhesions	6 (11.7)
No. prior abdominal surgeries (mean, SD)	2.2 ± 1.6
No. previous hernia repairs	
1	15 (29.4)
> 1	4 (7.9)
History mesh use	9 (17.6)
Hernia length (cm) (mean, SD)	11.9 ± 9.4
Hernia width (cm) (mean, SD)	8.1 ± 5.2
Hernia area (cm^2^) (mean, SD)	126.2 ± 153.5
Length of follow up (months, mean, SD)	12.8 ± 7.0

### Operative details

The average midline hernia width was 8.1 (± 5.2) cm. Preoperative botulinum toxin injection (300 units) injected three weeks prior to surgery was used in 11.8% of cases to improve abdominal wall compliance [17]. A total of 25.5% of patients required anterior component separation due to fascial tension that precluded primary closure without it. The most common mesh suture utilized was the “number 2 large needle” (50.8%) and the “number 1 large needle” (28.8%). In 12 cases (25.1%), mesh suture was additionally used in non-midline closures (ostomy site closures or parastomal hernia repairs). Secondary site outcomes are reported separately from the primary midline closure. The average hospital length of stay was 7.9 (± 6.1) days. The remaining operative details are in Table 2.

**Table 2:** Operative details

	*N* = 51 (%)
Length of inpatient stay (days) (mean, SD)	7.7 ± 6.0
Stoma present	19 (37.3)
Operative time (minutes)	328.3 ± 172.6
Pre-operative abdominal wall Botox	6 (11.8)
Anterior components separation	13 (25.5)
Mesh suture for second indication	
Ostomy site fascial closure	8 (15.7)
Parastomal hernia repair	4 (7.8)
Skin closure	
Suture	18 (35.3)
Staples	33 (64.7)

### Surgical site infections

The 1-year SSI rate was 15.7% (*N* = 8), with five infections requiring procedural or surgical intervention. One patient underwent mesh suture removal on postoperative day eight due to early fascial dehiscence and deep wound infection that required exploration. Operative findings noted an intact mesh suture knot with fascial deterioration and omental herniation and culture positive for *pseudomonas* and *enterococcus*. Two patients had reoperations for organ space infections from bowel anastomotic leaks; mesh suture was explanted in one case and replaced in the other. Two additional patients were treated with interventional radiology (IR) drainage of abdominal or pelvic fluid collections and antibiotics; infections resolved after treatment. No late or chronic mesh-related infections were observed. There were no SSIs at the secondary sites. No patient or operative factors were significantly associated with increased SSI risk on univariate analysis.

### Secondary outcomes

The 90-day SSO rate was 23.5% (*N* = 12) of patients and seven (13.7%) patients required procedural intervention (Table 3). Five of the interventions were described above under SSI, two additional were for surgical incision and drainage and interventional radiology aspiration of seromas. One patient developed recurrent enterocutaneous and urogenital fistulas following a complex index operation involving multiple fistula takedowns and a subsequent early anastomotic leak. The abdominal wall enterocutaneous fistulae were managed conservatively and were noted to have no output at 17 months postoperatively without surgical intervention. No chronic draining sinuses or mesh-related sinus tracts were observed. There was a single seroma documented at a secondary site, this was aspirated and resolved. No patient or operative factors were significantly associated with increased SSO risk on univariate analysis.

**Table 3:** Primary and secondary outcomes following mesh suture repair of clean contaminated and contaminated incisional hernias

	*N* = 51 (%)
SSI	
Superficial infection	2 (3.9)
Deep infection	2 (3.9)
Organ space infection	4 (7.8)
SSI 0–90 days	8 (15.7)
SSI 1 year	8 (15.7)
SSO	
SSI	8 (15.7)
Seroma	4 (7.8)
Hematoma	2 (3.9)
Soft tissue breakdown	1 (2.0)
Fascial dehiscence	1 (2.0)
Cellulitis	0 (0)
Suture granuloma	0 (0)
Chronic draining sinus	0 (0)
Enterocutaneous fistula	1 (2.0)
SSO 0–90 days*	12 (23.5)
SSO 1 year*	14 (27.5)
SSO requiring procedural intervention	7 (13.7)
Readmissions related to abdominal repair	3 (5.8)
Reoperations related to abdominal repair	5 (9.8)
Death 1-year	3 (5.9)
Hernia recurrence	4 (8.2)
Length of time hernia recurrence (mo.) (mean ± SD)	12.9 ± 7.3
Method of determining hernia recurrence	
Clinical exam	32 (62.7)
Radiographic	17 (33.3)

*Represents the number of patients with at least one SSO. If a patient experienced multiple SSO-related complications, they were counted as a single SSO in this total. All individual events are detailed separately within the table.

Readmissions occurred in 17.6% of patients within 90 days and 27.5% within one year. However, only three readmissions were related to the index surgery: a superficial SSI (managed with IR drainage), bowel obstruction (managed non-operatively), and a delayed bowel leak (managed non-operatively). A total of five patients (9.8%) underwent reoperation within one year. Three early reoperations (< 90 days) were for bowel perforation, anastomotic leak, and fascial dehiscence with deep infection, described previously. In all three cases, the index mesh suture was divided and removed to access the peritoneal cavity. In one case, new running mesh suture was used for fascial re-approximation. In the remaining two cases, the abdominal wall was reconstructed with either interrupted strips of polypropylene mesh or an underlay of Vicryl mesh. Two late reoperations (91 to 365 days) included one hernia recurrence repair, and one extra-abdominal ostomy prolapse. The recurrent hernia was repaired with an intraperitoneal placement of bilaminar synthetic mesh (polypropylene mesh with ePTFE barrier).

### Hernia recurrence

During follow-up, four hernia recurrences (8.2%) were documented. Three occurred within 18 months and one at 22.8 months. Two recurrences were confirmed by CT scan, while two were documented on clinical examination. Recurrence occurred as early as 5.6 months postoperatively. The mean time to recurrence was 12.8 (± 7.3) months. Two of the four recurrences underwent reoperation. One measured 2 × 2 cm, located between two loops of the running mesh suture, and was repaired with repeat mesh suture. The second measured 10 cm transversely; although the mesh suture remained intact, the fascia had separated and was repaired with intraperitoneal planar permanent mesh and absorbable suture. Of the two unrepaired recurrences, one appeared on clinical exam five months after an intervening minimally invasive ostomy reversal where no hernia had been noted. The other was incidentally identified on CT scan at an outside hospital performed for unrelated reasons.

**Fig. 4:**
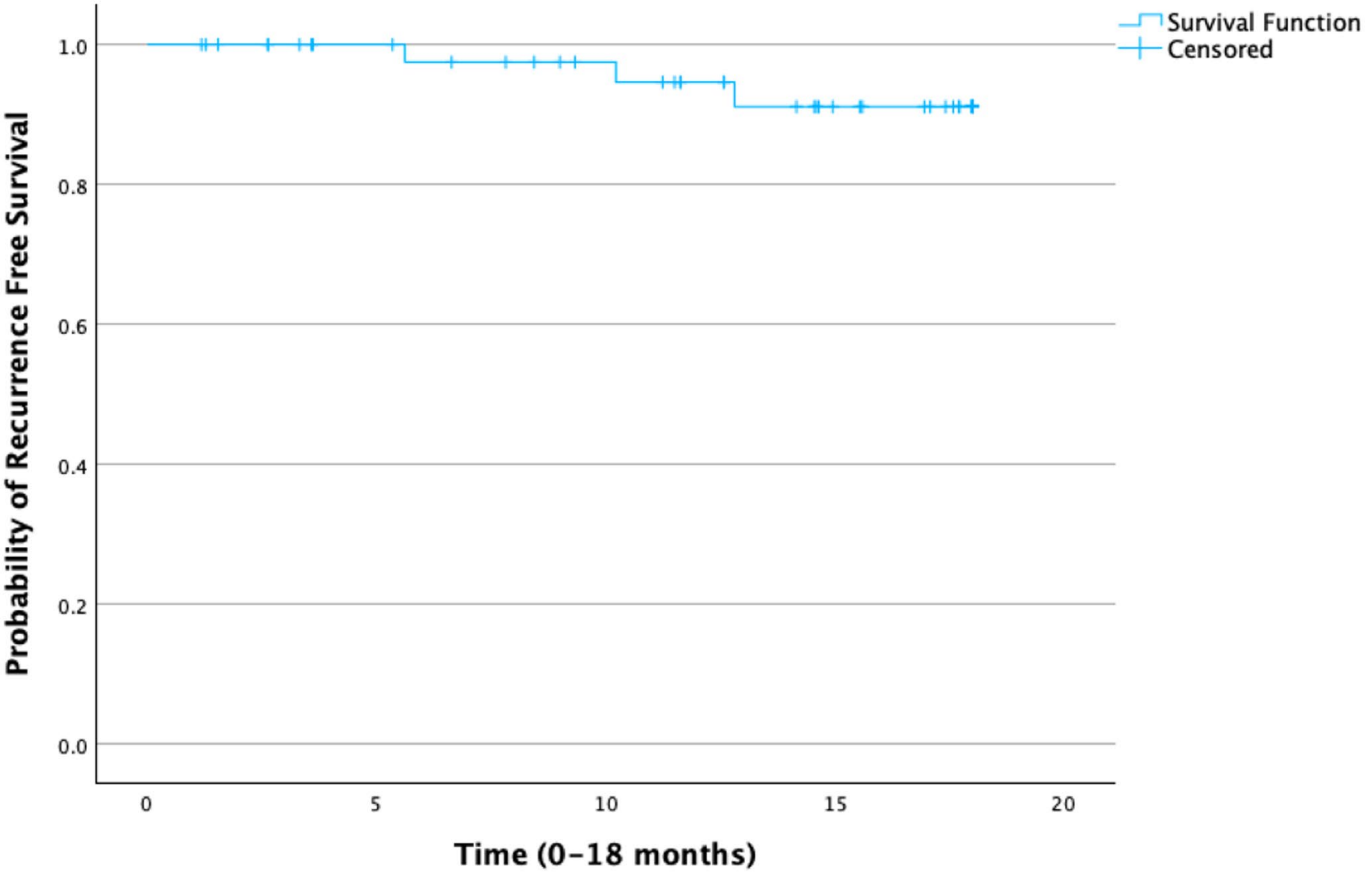
Kaplan-Meier curve demonstrating recurrence-free survival truncated at 18 months. Three hernia recurrences occurred during the truncated follow-up period. An additional recurrence that occurred after 18 months is not shown on this curve but is included in the overall event count. Vertical ticks represent censored observations (patients with no recurrence by last follow-up)

Three patients died within one year and one during extended follow-up, from causes unrelated to index surgery or hernia repair. Two patients had mesh suture explanted during reoperation for early bowel leak (< 30 days) and were excluded from long-term recurrence analysis. Kaplan-Meier analysis demonstrated a 12-month recurrence-free survival of approximately 91% (Fig. 4). The estimated mean recurrence-free survival was 17.3 months (95% CI: 16.5–18.1). Median recurrence-free survival was not reached due to the low number of events. The survival curve was truncated at 18 months due to limited long-term follow-up beyond that point. One recurrence occurring after 18 months was not included in the curve. No recurrences occurred in patients who received mesh suture for non-midline closure (ostomy site or parastomal hernia).

### Major complications

Major complications (defined as SSI, SSE, or reoperation within 90 days) occurred in 11 patients (21.6%) and presence of a stoma at the time of hernia repair was the only significantly associated variable (χ² = 4.176, df = 1, *p* = 0.041). (Table 4)

**Table 4:** Patient preoperative demographics, comorbidities, and surgical details associated with major complications

	No Major complications	Complication or reoperation 0–90 days	*p*-value
	*N* = 40	*N* = 11	
Age (mean, SD)	62.8 ± 13.8	61.2 ± 16.2	0.75
Gender			
Male	14 (35.0)	7 (63.6)	0.087
BMI (mean, SD)	28.6 ± 7.0	29.7 ± 9.3	0.481
ASA Class			0.669
II	3 (7.5)	1 (9.1)	
III	34 (85.0)	10 (90.9)	
IV	3 (7.5)	0 (0)	
Wound Class			0.525
Clean contaminated	26 (65.0)	6 (54.5)	
Contaminated	14 (35.0)	5 (35.5)	
Active smoking	3 (7.5)	2 (18.2)	0.291
Former smoking	19 (47.5)	7 (63.6)	0.343
Cancer	12 (30.0)	3 (27.3)	0.860
COPD	9 (22.5)	1 (9.1)	0.321
HTN	32 (80.0)	8 (72.7)	0.603
DM	10 (25.0)	2 (18.2)	0.637
Preoperative abdominal wall Botox	4 (10.0)	2 (18.2)	0.456
Anterior components release	11 (27.5)	2 (18.2)	0.530
Source of contamination			0.571
Bowel resection	18 (45.0)	4 (36.4)	
Ostomy reversal	3 (7.5)	0 (0)	
Gynecologic procedure	3 (7.5)	1 (9.1)	
Urologic procedure	3 (7.5)	1 (9.1)	
Creation of stoma/revision	2 (5.0)	2 (18.2)	
Enterotomy	1 (2.5)	0 (0)	
Cholecystectomy	2 (5.0)	0 (0)	
Fistula	3 (7.5)	2 (18.2)	
Lysis of adhesions	5 (12.5)	1 (9.1)	
**Stoma present**	**12 (30.0)**	**7 (63.6)**	**0.041**
No. abdominal surgeries (mean, SD)	2.2 ± 1.5	2.6 ± 2.1	0.514
No. hernia repairs (mean, SD)	0.43 ± 0.68	0.64 ± 0.81	0.383
Operative time (min) (mean, SD)	324.6 ± 187.2	341.8 ± 109.3	0.772
Duration of stay (days) (mean, SD)	6.8 ± 4.7	11.1 ± 8.9	0.149

## Discussion

This study suggests that mesh suture may be a feasible and generalizable technique for midline hernia repairs in contaminated and clean contaminated fields. Among 51 patients treated across an integrated health system, short-term outcomes included a 90-day SSI rate of 15.7% and SSO rate of 23.5%. Observed complication rates are consistent with those reported in the literature for biologic, biosynthetic, and permanent synthetic meshes in similar settings, where 90-day SSI rates range from 15 to 24% and SSO rates of 25–48% and suture only repairs, with SSI rates of 13–19% [3–6, 18, 19]. No chronic mesh-related infections or persistent drainage were observed. Notably, mesh suture was used by 22 surgeons across seven specialties, most without formal abdominal wall reconstruction training, highlighting its feasibility across diverse surgical backgrounds.

### Suture only repair

Mesh suture represents a novel alternative for the closure of contaminated abdominal wall defects, where traditional options such as monofilament suture and planar mesh may carry unacceptable risks or limitations. Traditional sutures, including slowly absorbable monofilaments like polydioxanone (PDS) and polyglyconate (Maxon), remain commonly used in contaminated field closures. However, suture-only repairs are associated with high rates of fascial dehiscence and hernia recurrence, particularly under high-tension conditions, with historical hernia recurrence approaching 50% in some series [20, 21].

While limited data exists specifically for suture-only closure of contaminated hernias, outcomes from contaminated laparotomy offer a useful clinical reference. Reported dehiscence rates in this setting range from 2.6 to 3.9%, with even higher rates seen in contaminated emergency cases closed with barbed suture (5.7%) [22–24]. In the present study, only one patient (2.0%) experienced fascial dehiscence, despite the added tension associated with concurrent hernia repair, suggesting favorable mechanical performance relative to historical benchmarks. Regarding hernia recurrence, a recent study of 569 patients undergoing ileostomy site closure with sutures (56.8% polydioxanone (PDS), 32.3% polyglyconate (Maxon), 1.9% polyglactin (Vicryl), 3.2% polypropylene) found a one-year incisional hernia rate of 35.7% [25]. In contrast, the one-year clinical hernia rate in this mesh suture cohort was 8.2%. The mechanical advantage of mesh suture may lie in its ability to flatten under tension, increasing contact points and distributing load across a broader surface, which may lower the risk of pull-through failure. While slowly absorbable sutures minimize foreign body burden, they may not generate a long-term scar response that is required to achieve a lasting closure. Mesh suture, as a permanent scaffold, remains in place throughout healing and may contribute to sustained support. These results suggest that mesh suture may offer meaningful improvements over conventional suture-only closure in contaminated fields.

Infection-related complications are also critical to consider. The SSI rate of 15.6% in this study is consistent with large series of open contaminated laparotomy closures, which range from 13 to 19% depending on the surgical context [24]. Notably, there were no cases of sinus formation in this study, despite the use of permanent, bonded polypropylene filaments. The expected chronic sinus rate of a permanent suture used in abdominal wall closures is 3.5% [22]. Mesh suture’s rapid fibrovascular incorporation, smaller filament diameter, and widely spaced braid structure may all contribute to reduced bacterial colonization and thus, infectious complications [26, 27]. If a sinus were to develop, the foreign material would be located immediately under the skin incision to facilitate excision.

### Planar mesh augmentation

The limitations in traditional suture-only repairs established the foundation for mesh reinforcement in incisional hernia repair. In contaminated fields, biologic mesh was initially favored due to its presumed resistance to infection. However, long-term durability has been disappointing, with recurrence rates frequently exceeding 30% and SSI rates reaching 25–30% across multiple series [3, 5, 28]. In a prospective study of single-stage biologic mesh repair, Rosen et al. reported a 19% SSI rate, 5% mesh explant rate, and 29% recurrence rate [29]. Comparative trials and meta-analyses further highlighted the inferior performance of biologics relative to synthetic mesh, with recurrence rates as high as 42% and consistently higher wound morbidity [13, 18].

In response, biosynthetic, or slowly absorbable mesh such as poly-4-hydroxybutyrate, have emerged as a potentially promising alternative. Early studies in contaminated fields reported SSI rates between 13 and 24% and mesh explant rates below 5%, suggesting more favorable infection profiles relative to biologics [6, 8, 18, 30, 31]. For example, Amro et al. retrospectively compared onlay biosynthetic mesh repair versus underlay biologic hernia repair in contaminated settings. They found both lower SSO rates (31.1% versus 47.8%) and SSI rates (13.3% versus 23.9%) for biosynthetic mesh compared to biologic [8]. These findings suggest that biosynthetic meshes may offer greater infection tolerance comparable to biologics, and possible improved durability with recurrence rates reported between 15 and 22% at 12–24 months [4, 6, 8].

Permanent synthetic mesh has traditionally been avoided in contaminated settings due to concerns about chronic infection, fistula formation, and explantation. However, contemporary literature now supports its selective use in contaminate fields. Carbonell et al. reported outcomes from 133 contaminated ventral hernia repairs using lightweight, polypropylene mesh placed in the retrorectus position, observing an SSI rate of 21%, mesh explantation rate of 4% and hernia recurrence rate of 15.3% [7]. Similarly, Rosen et al. conducted a randomized controlled trial comparing biologic versus synthetic mesh in single-stage repairs of clean contaminated and contaminated hernias. At two years, synthetic mesh had significantly lower recurrence rates (5.6% versus 20.5%), no significant increase in mesh-related complications, and significantly lower implant cost [5]. Majumder et al. found that synthetic mesh had a lower recurrence rate (8.9% versus 26.3%) and reduced wound morbidity compared to biologic mesh in clean contaminated cases as well [13]. A large meta-analysis by Rodriguez-Quintero et al. similarly concluded that permanent synthetic mesh outperformed biologic and absorbable alternatives in both infection complications and recurrence; specifically, this study documented the 1–year contaminated hernia recurrence rate for 1823 patients to be 23% for permanent synthetics and this may be a more realistic “real-world” outcome for synthetic material used in contaminated hernia repairs [18].

Despite these favorable outcomes for synthetic mesh in contaminated hernia repairs, planar mesh placement remains technically demanding and may not be feasible in all clinical scenarios. In 2018, Dumanian et al. proposed a novel technique using narrow strips of polypropylene mesh applied in a suture-like fashion. Among 48 clean contaminated, contaminated, and dirty cases, this approach yielded a 19% SSI rate, 27% SSO rate, and 13% recurrence rate at 12 months with no mesh explants [32]. This concept laid the foundation for the applicability of mesh suture for incisional hernia repair in non-clean environments.

The 90-day SSI rate in this mesh suture study was 15.7%, within the expected range for planar mesh repairs in contaminated fields of 15–24% [3–6, 32]. Only three SSIs in this series could directly be attributed to the mesh suture; the remaining cases involved deep or organ space infections unrelated to the fascial closure. One case required reoperation for a deep space infection with fascial dehiscence, where the suture was easily divided and excised along the fascial edge. In such cases that require reoperation, the placement of mesh suture along the fascial edge, immediately beneath the subcutaneous tissue, allows for straightforward identification and removal with substantially less dissection than is typically required for planar mesh removal. This may represent a practical advantage in managing mesh-related complications when reoperation is necessary. The 90-day SSO rate was 23.5%, below published ranges of 25–43% for contaminated repairs using planar mesh [3–6, 32]. There were no instances of mesh infections requiring explanation. Although mesh suture is composed of permanent polypropylene filaments, mesh suture has markedly lower foreign body burden as compared to planar mesh (0.53–0.96 g for a 30 cm closure versus 15.7–20.3 g for a 450 cm² mesh implant), which may reduce infection risk [33]. Additionally, preclinical data support that mesh suture undergoes rapid fibrovascular incorporation, as early as eight days, potentially limiting risk of chronic-mesh related infections [12].

One patient (2.0%) in this series developed a recurrent enterocutaneous fistula during follow-up. Although mesh suture introduces a greater surface area and foreign material load than standard monofilament suture, this incidence remains within the expected range reported for contaminated closures of up to 3.5% [22]. In this case, the fistula occurred in the context of a highly complex surgical history, including multiple prior urogenital fistulas and an early anastomotic leak. When conceptualized anatomically, mesh suture forms a loop that transverses fascia and muscle, with only a small arc entering the peritoneal cavity. In re-operative or scarred fields, it is often not feasible to completely exclude the peritoneum from the fascial bite, and this may result in transient proximity of foreign material to inflamed bowel or anastomotic sites. While braided suture is well known to be associated with higher risk of bacterial colonization and biofilm formation, mesh suture differs from traditional braided sutures in filament configuration. While mesh suture is indeed a braid, the widely separated filaments act more like a collection of monofilaments rather than a braided suture where bacteria can potentially hide in the interstices [27]. Nonetheless, the potential for biofilm formation remains a valid concern, particularly in contaminated fields. Classical surgical principles discourage placing foreign material on compromised bowel for this reason. Although registry data on mesh suture use in abdominal wall repair (*N* = 862) suggests a low fistula rate (0.2%), this outcome warrants continued surveillance [34]. We also acknowledge that the true incidence of both enterocutaneous fistula and chronic draining sinus formation may be underestimated in this small series. Long-term follow-up with large cohorts will be essential to better understand these risks.

It is well known that contaminated incisional hernia recurrence rates are high with one-year data from the Abdominal Core Health Quality Collaborative (ACHQC) documenting recurrence rates of 23% for permanent mesh, 40% for absorbable mesh, and 32% for biologic mesh [18]. Hernia recurrence in our study was observed in four patients (8.2%), with three occurring within the 18-month follow-up window. Kaplan-Meier analysis showed a 12-month recurrence-free survival of approximately 91%, with a mean recurrence-free survival of 17.3 months (95% CI: 16.5–18.1). While these results are promising, definitive conclusions about long-term durability are not possible due to limited follow-up. Importantly, if recurrence does occur, the use of mesh suture at the index procedure preserves favorable anatomy for subsequent retrorectus mesh abdominal reconstruction.

### Limitations

This study was not designed to be a comparative trial, and the absence of a control group limits the ability to draw causal inferences. Additional limitations include the retrospective design, relatively short follow-up period, and lack of standardization in perioperative management protocols (use of closed-suction drains, timing and duration of antibiotic therapy), all of which may influence the rates of SSI and SSOs. Nearly half of the surgeries were performed by a single surgeon (G.A.D), which may improve internal consistency in technique but introduces operator technical skills as a confounding variable. However, the inclusion of patients treated by 21 additional surgeons across academic and community settings supports the potential for broader surgeon adoption and greater generalizability across patient populations. Future prospective and multicenter trials with longer follow-up are needed to establish long-term durability and comparative effectiveness.

## Conclusion

Mesh suture closure in clean contaminated and contaminated midline incisional hernia repair appears feasible and reproducible, with short-term complication and hernia recurrence rates that are encouraging when compared to published outcomes for standard suture, biologic, biosynthetic, and permanent mesh repairs. The simplicity of use and avoidance of additional tissue plane dissection is a benefit in comparison to planar meshes. As such, mesh suture may represent a feasible alternative in the care of these patients. Prospective, comparative studies are warranted to further define its role in contaminated abdominal wall hernia repair and reconstruction.

## Electronic supplementary material

Below is the link to the electronic supplementary material.

Supplementary Material 1

Supplementary Material 2

Supplementary Material 3

Supplementary Material 4

Supplementary Material 5

Supplementary Material 6

Supplementary Material 7

Supplementary Material 8
